# Jailed in the mountains: Genetic diversity and structure of an endemic newt species across the Pyrenees

**DOI:** 10.1371/journal.pone.0200214

**Published:** 2018-08-02

**Authors:** Emilio Valbuena-Ureña, Neus Oromi, Anna Soler-Membrives, Salvador Carranza, Fèlix Amat, Sebastià Camarasa, Mathieu Denoël, Olivier Guillaume, Delfí Sanuy, Adeline Loyau, Dirk S. Schmeller, Sebastian Steinfartz

**Affiliations:** 1 Unitat de Zoologia, Facultat de Biociències, Universitat Autònoma de Barcelona, Cerdanyola del Vallès (Barcelona), Catalonia, Spain; 2 Centre de Fauna Salvatge de Torreferrussa (Catalan Wildlife Service–Forestal Catalana), Barcelona, Catalonia, Spain; 3 Departament de Ciència Animal (Fauna Silvestre), Universitat de Lleida, Lleida, Catalonia, Spain; 4 Institute of Evolutionary Biology (CSIC-Universitat Pompeu Fabra), Barcelona, Spain; 5 Àrea d’Herpetologia, Museu de Granollers, Ciències Naturals, Granollers, Catalonia, Spain; 6 Laboratory of Fish and Amphibian Ethology, Behavioural Biology Group, Freshwater and OCeanic science Unit of reSearch (FOCUS), University of Liège, Liège, Belgium; 7 Station d'Ecologie Théorique et Expérimentale CNRS-Université de Toulouse, Moulis, France; 8 Helmholtz Centre for Environmental Research–UFZ, Department of Conservation Biology, Leipzig, Germany; 9 EcoLab, Université de Toulouse, CNRS, INPT, UPS, Toulouse, France; 10 Zoological Institute, Department of Evolutionary Biology, Unit of Molecular Ecology, Technische Universität Braunschweig, Braunschweig, Germany; University of British Columbia Okanagan, CANADA

## Abstract

The Pyrenees represent a natural laboratory for biogeographic, evolutionary and ecological research of mountain fauna as a result of the high variety of habitats and the profound effect of the glacial and interglacial periods. There is a paucity of studies providing a detailed insight into genetic processes and better knowledge on the patterns of genetic diversity and how they are maintained under high altitude conditions. This is of particular interest when considering the course of past climate conditions and glaciations in a species which is considered site tenacious, with long generation times. Here we analyzed the genetic patterns of diversity and structure of the endemic Pyrenean brook newt (*Calotriton asper*) along its distribution range, with special emphasis on the distinct habitat types (caves, streams, and lakes), and the altitudinal and geographical ranges, using a total set of 900 individuals from 44 different localities across the Pyrenean mountain range genotyped for 19 microsatellite loci. We found evidence for a negative longitudinal and positive altitudinal gradient of genetic diversity in *C*. *asper* populations. The fact that genetic diversity was markedly higher westwards is in accordance with other Pyrenean species. However, the impact of altitudinal gradient on the genetic diversity seems to differ from other species, and mostly from other amphibians. We found that lower altitudes can act as a barrier probably because the lowlands do not provide a suitable habitat for *C*. *asper*. Regarding the distinct habitat types, caves had significantly lower values of genetic diversity compared to streams or lakes. The mean *F*_ST_ value was relatively high (0.304) with maximum values as high as 0.771, suggesting a highly structured total population. Indeed, populations were grouped into five subclusters, the eastern populations (cluster 1) remained grouped into two subclusters and the central-western Pyrenees (cluster 2) into three subclusters. The increase of isolation with geographical distance is consistent with the population structure detected. In conclusion, *C*. *asper* seems to be adapted to high altitude mountain habitats, and its genetic diversity is higher in the western Pyrenees. In terms of conservation priority, we consider more relevant the populations that represent a reservoir of genetic diversity.

## Introduction

Past and contemporary climate conditions have been the main drivers shaping the genetic population structure of species [1–3]. Across the various mountain ranges in Europe, glacial and postglacial periods have forced many species to go through severe processes of contraction and expansion, leading to repeated occurrences of colonization or recolonization. These range fluctuations form the basis of their current geographic distribution as well as their population genetic structure [1]. Generally, climatic conditions in mountains are extreme, representing a considerable selection force, frequently leading to local extinction of populations. Also climate change has an important impact on mountain ecosystems by increasing the frequency of extreme weather events, such as high temperature variability, changes in seasonality, and variability in precipitation with various impacts on species populations [4, 5]. Therefore, mountain ranges are excellent areas to study the process of repeated colonization events after harsh climatic conditions, e.g. glacial periods during which many species have gone locally extinct or populations suffering from small population sizes as a consequence of unfavorable environmental conditions [6, 7].

The Pyrenees have played an important role throughout the distinct climatic events acting as a barrier or as a bridge for the migration of species between Europe and the Iberian Peninsula. During glacial periods, the ice sheet rarely descended below 1000 m above sea level (asl) but did not cover the highlands completely [2, 8]. These unglaciated areas could have provided refugia for some species that had to escape from the glaciated high elevation parts. Recent studies, such as on spiders (*Harpactocrates ravastellus;* [9]), butterflies (*Erebia epiphron*; [10]) and plants (*Rhododendron ferrugineum*; [8]) proposed two main refugia, one in the western-central Pyrenees and one in the eastern Pyrenees. More or less severe bottlenecks for populations are assumed, with successive range expansions from these refugia when the temperature increased during interglacial periods (e.g. last 100,000–10,000 years ago (ya); [11, 12]. These historical processes had a strong impact on shaping the complex genetic population structure of many species in the Pyrenees. Additionally, the Pyrenees provide a great variety of different habitats and environments, such as caves, high altitude lakes or streams [6, 7]. Contemporary gradients, such as altitude or longitude, play an important role in the Pyrenees, as they shape the existing proportion of suitable habitats along the mountains [13, 14]. Longitude is an important gradient to take into account because the influence of the Atlantic Ocean provides a cooler and wetter climate westwards than in eastern areas, which are more influenced by the Mediterranean temperate climate [8]. Habitats, comprising lakes (mostly of glacial origin), caves or streams, can be found along an elevational gradient from lowlands and foothills up to 3,400 m asl. These gradients provide a variety of habitats suitable for colonization but some are sufficiently different that local adaptation may need to occur to allow for successful establishment of populations after colonization. Hence, the variety of habitats may also have an impact on the genetic population structure by promoting genetic divergence of differentially adapted local genotypes [15–19].

Amphibians are excellent models to explore patterns of gene flow and genetic structure due to their strong habitat-association which is correlated with water availability [20]. Besides water availability, the genetic structure of an amphibian species is also strongly influenced by its dispersal capacity [21, 22]. Amphibians with high dispersal rates show poorly structured metapopulations (e.g. *Epidalea calamita*, [23]; *Rana temporaria* and *B*. *bufo*, [24]; *R*. *arvalis*, [25]). Instead, highly phylopatric amphibian species with very restricted dispersal rates (e.g. *Calotriton arnoldi*) present highly structured and isolated populations [26].

Here we use the endemic Pyrenean amphibian species *Calotriton asper* as a case study to better understand the role of climatic events and habitat variability in shapping the genetic structure of a mountain species. The Pyrenean brook newt, *C*. *asper*, inhabits different habitat types such as streams, alpine lakes and caves at elevations ranging from 360 m asl to 3,000 m asl across the Pyrenees and the pre-Pyrenean mountain chain occupying a geographic distribution range of more than 20.000 km^2^ [27, 28]. *Calotriton asper* is classified as near threatened by the International Union for Conservation of Nature [27, 28]. Although this species occupies different environments, its optimal habitats are fast-flowing streams at elevations between 750–1500 m asl with a strong slope and water temperatures not exceeding 15–17°C during summer. These habitats are characterized by abundant riverbank vegetation, which helps to prevent the heating of water above the thermal limits of this species [28]. High mountain lakes constitute secondary habitats for *C*. *asper*. In these lakes, *C*. *asper* may sometimes be located in the vicinity of the entrance of small streams or in the areas of drainage, where the oxygen content of the water is high [28]. Clergue-Gazeau and Martínez-Rica [29] distinguished a third group of habitats formed by underground courses, upwellings and sources. Such cave habitats may exhibit distinct ecological characteristics and aspects, although they are in some way reminiscent of the usual habitat. For *C*. *asper*, a 2-year-long terrestrial dispersal phase is described during its juvenile stage following metamorphosis [30], but it is unclear how far individuals do disperse at this stage. Previous capture-mark-recapture studies [31] suggested that the dispersal capability of adult newts is limited (less than 50 m per year). On a broader geographic scale, analysis of the genetic population structure will also allow inferences on dispersal and movement between populations.

Previous studies on the Pyrenean brook newt examined its genetic population structure and phylogeography with different molecular markers. The mitochondrial sequence variation of the cytochrome *b* gene revealed low levels of genetic variation and poorly structured haplotype networks (two main mt-haplotypes with a star-like shape)[11, 32, 33]. These results suggest a rather recent recolonization after the last glacial maximum (LGM), which roughly ended in the Pyrenees around 15,000–10,000 ya [34]. Isolated populations that remain from glacial refugia retained the genetic diversity, which was fixed in a few mtDNA haplotypes. During the following warmer climate period an altitudinal habitat switch occurred: lower elevations, that have been suitable habitats during the glacial period, became too warm and unsuitable, while previously unsuitable areas during the LGM (habitats at high altitudes in the Pyrenees) became suitable niches and were presumably colonized from few source populations with small population sizes [3, 35]. It is expected that species that followed this common pattern exhibit relatively low levels of genetic diversity. However, in contrast to this general pattern, high levels of genetic diversity and differentiation among localities have been reported by Milá *et al*. [32] using nuclear AFLP loci reflecting a more genome-wide pattern of genetic differentiation. However, this study was unevenly biased towards the French side of the Pyrenees (17 localities), not covering more than half of the distribution range of this species on the Spanish side. Furthermore, *C*. *asper* populations include hypogean and epigean habitats, exhibiting different mating preferences [36], life-history traits [37] and metabolism [38]. Further, AFLP loci are of limited use for genetic analysis as loci are anonymous and co-dominant and therefore homozygotes cannot be differentiated from heterozygotes. We therefore apply here microsatellite loci in combination with a range-wide sampling of this species.

Given the limitation of AFLPs and contrasting patterns by marker type, here we analyze the current genetic patterns of diversity and structure of the Pyrenean brook newt *Calotriton asper* along its entire distribution area covered by 44 sampling localities across the Pyrenean mountain chain and based on 19 species-specific microsatellite loci. Our study aims to (i) characterize the genetic diversity of each *C*. *asper* population, (ii) analyze the genetic structure among its populations, (iii) determine whether the genetic patterns are related to a longitudinal and altitudinal gradient or to distinct habitat types, and (iv) whether mountains constitute a natural barrier between French and Iberian *C*. *asper* populations.

## Materials and methods

### Ethics statement

The collection of all samples was conducted under the licenses required by the corresponding authorities. As for the Spanish samplings, permits were given by the following institutions: Departament d’Agricultura, Ramaderia, Pesca Alimentació i Medi Natural of the Catalan Government, with the permission numbers SF/90, SF/91 and SF/429; Servicio de Conservación de la Biodiversidad, Departamento de Desarrollo Rural y Medio Ambiente of the Navarra Government, with the permission number 2012/721; Instituto Aragonés de Gestión Ambiental, Area II-Biodiversidad of Aragon Government, with the permission numbers 24/2009/4323, 24/2010/901, 24/2012/661 and 24/2014/491. As for the French samplings, the present investigation was carried out according to the ethical principles of the French (Ministère de l'Agriculture) and European Convention for the Protection of Vertebrate Animals Used for Experimental and Scientific Purposes (Council of Europe, no. 123, Strasbourg, 1985) at the Station of Experimental Ecology of Moulis, France (Arrêtés 2009–11 for Haut Garonne and 2009–19 for Ariège).

All tissue samples were collected according to the requirements of the above administrative institutions: newts were captured manually and tissue samples were taken with a procedure which does not affect survival and body condition in newts [39]. Immediately after the completion of the procedure, tissue samples were stored in absolute ethanol. No individuals were severly harmed or sacrificed.

### Field sampling and microsatellite loci genotyping

**Fig 1:**
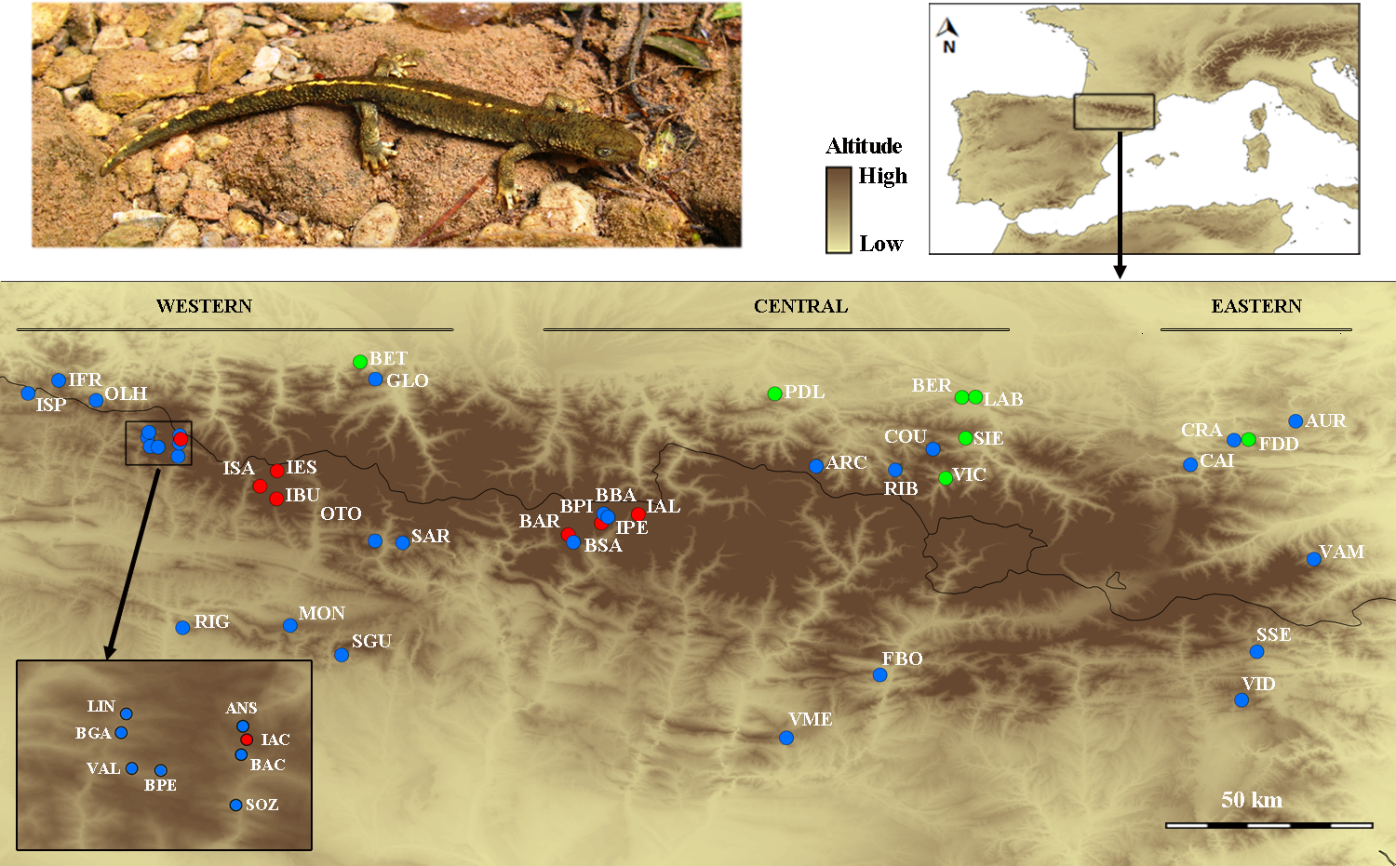
Location of the sampled localities of *Calotriton asper* populations. Location of the sampled localities of *Calotriton asper* populations (the colors correspond to the distinct habitat types; blue: streams; green: caves; red: lakes). See Table 1 for details on each locality.

A total of 900 individuals of *C*. *asper* were analyzed from 44 different localities covering most of its distribution range (Fig 1, Table 1). Tissue samples consisted of small tail or toe clips preserved in absolute ethanol. Genomic DNA was extracted using the Qiagen (Valencia, CA, USA) DNeasy Blood and Tissue Kit, following the manufacturer’s protocol. Individuals were genotyped for a total set of 19 microsatellite loci. PCR conditions and genotyping of loci were as in Drechsler *et al*. [40]

**Table 1:** Geographical information and genetic diversity for *Calotriton asper* populations. Geographical information and estimates of genetic diversity parameters for each *Calotriton asper* population and cluster defined by STRUCTURE analysis. Altitude in meters; N, sample size; Na: number of alleles per locus; Ar, allelic richness; PA, number of private alleles; PAAr, allelic richness of private alleles; *F*_IS_, inbreeding coefficient; H_O_, observed heterozygosity; H_E_, expected heterozygosity. Values in bold indicate statistical significance after Bonferroni’s correction.

Grouping	Code	Long	Lat	Alt	Habitat	N	Na	Ar	PA	PAAr	H_O_	H_E_	*F*_IS_
Population													
Irati Spain	ISP	-1.14	43.01	848	Stream	16	4.895	3.210	1	0.020	0.576	0.596	0.067
Irati France	IFR	-1.06	43.05	1105	Stream	15	4.421	3.080		0.010	0.603	0.572	-0.019
Olhadoko	OLH	-0.95	42.99	662	Stream	15	4.474	3.050		0.010	0.630	0.572	-0.068
Barranco Gamuetta	BGA	-0.80	42.89	1324	Stream	50	7.000	3.500		0.030	0.609	0.633	0.049
Linza	LIN	-0.80	42.90	1374	Stream	39	6.632	3.490	1	0.030	0.606	0.621	0.037
Valdagras	VAL	-0.79	42.86	1266	Stream	39	6.000	3.400		0.020	0.641	0.619	-0.022
Barranco de Petraficha	BPE	-0.77	42.86	1427	Stream	21	6.263	3.680		0.020	0.644	0.650	0.034
Selva de Oza	SOZ	-0.71	42.83	1181	Stream	21	5.789	3.590		0.010	0.642	0.656	0.047
Barranco de Acherito	BAC	-0.71	42.87	1385	Stream	6	4.158	3.540		0.010	0.675	0.615	-0.007
Ansabere	ANS	-0.71	42.89	1787	Stream	20	4.368	3.020		0.010	0.579	0.573	0.016
Ibón de Acherito	IAC	-0.71	42.88	1882	Lake	44	6.316	3.260	1	0.030	0.595	0.598	0.017
Riglos	RIG	-0.70	42.34	920	Stream	4	3.000	3.000		0.100	0.461	0.487	0.219
Ibón de Saman	ISA	-0.48	42.74	2143	Lake	20	4.421	2.900	1	0.060	0.535	0.539	0.034
Ibón de Bucuesa	IBU	-0.43	42.71	2126	Lake	19	3.158	2.440	1	0.090	0.460	0.449	0.002
Ibón de Espeluchieca	IES	-0.43	42.79	1966	Lake	20	4.526	2.930		0.000	0.563	0.528	-0.040
Montrepos	MON	-0.39	42.34	1220	Stream	26	7.947	4.450	10	0.400	0.691	0.777	0.131
Sierra de Guara	SGU	-0.25	42.26	975	Stream	18	6.368	3.920	7	0.380	0.684	0.689	0.036
Betharram	BET	-0.19	43.10	451	Cave	31	1.579	1.440		0.000	0.182	0.182	0.016
Genie Longue	GLO	-0.15	43.05	671	Stream	16	3.368	2.280		0.000	0.408	0.388	-0.020
Oto	OTO	-0.15	42.59	1137	Stream	18	7.263	4.120	4	0.180	0.712	0.711	0.028
Sarvisé	SAR	-0.07	42.58	1359	Stream	21	8.211	4.500	4	0.230	0.723	0.764	0.078
Barbarisa	BAR	0.41	42.61	2333	Lake	12	4.684	3.360		0.090	0.605	0.609	0.049
Barranco de Sabaril	BSA	0.42	42.58	1684	Stream	20	5.316	3.280	1	0.040	0.597	0.601	0.032
Ibón de Perramó	IPE	0.50	42.64	2406	Lake	69	4.053	2.510		0.010	0.448	0.457	0.028
Barranco de Batisielles	BBA	0.51	42.67	1815	Stream	6	3.737	3.270		0.020	0.553	0.564	0.111
Barranco del Pino	BPI	0.52	42.66	1604	Stream	15	4.421	3.080		0.010	0.544	0.569	0.078
Ibón de Alba	IAL	0.61	42.66	2301	Lake	64	4.684	2.690	3	0.080	0.495	0.505	0.028
Pas du Loup	PDL	1.00	43.01	489	Cave	18	1.737	1.480		0.000	0.184	0.179	-0.001
Vilanova de Meià	VME	1.03	42.02	847	Stream	19	3.368	2.500	1	0.120	0.483	0.452	-0.039
Font Bordonera	FBO	1.30	42.20	748	Stream	20	3.632	2.330		0.010	0.387	0.386	0.023
Arcouzan	ARC	1.12	42.80	1214	Stream	8	3.895	3.280		0.000	0.704	0.620	-0.069
Ribaui	RIB	1.34	42.79	802	Stream	13	4.316	3.200		0.030	0.636	0.600	-0.019
Courbiere	COU	1.45	42.85	1626	Stream	6	3.211	2.860		0.000	0.579	0.542	0.024
Vicdessos	VIC	1.49	42.77	725	Cave	7	3.158	2.650	1	0.030	0.549	0.480	-0.068
Siech	SIE	1.55	42.88	691	Cave	8	2.421	2.150	1	0.030	0.375	0.380	0.079
Bernard	BER	1.53	43.00	565	Cave	26	2.579	1.990		0.000	0.366	0.353	-0.019
Labouiche	LAB	1.57	43.00	485	Cave	17	2.947	2.420		0.010	0.483	0.468	0.000
Cailla	CAI	2.19	42.81	724	Stream	23	3.000	2.190		0.010	0.455	0.409	-0.030
Cass Rats	CRA	2.32	42.88	522	Stream	22	1.947	1.660		0.000	0.282	0.245	-0.127
Font de Dotz	FDD	2.36	42.88	485	Cave	4	1.842	1.840	1	0.050	0.434	0.289	-0.385
Auriac	AUR	2.49	42.93	543	Stream	7	1.789	1.700		0.000	0.293	0.269	-0.013
Vidrà	VID	2.34	42.13	1068	Stream	14	6.526	4.030		0.040	0.473	0.710	0.367
St Pau de Segúries	SSE	2.38	42.27	892	Stream	13	2.684	2.110		0.030	0.356	0.354	0.034
Valmanya	VAM	2.54	42.53	924	Stream	10	2.421	2.000		0.000	0.358	0.318	-0.075
Clusters													
Cluster 1						479	13.842	13.700	72	5.998	0.582	0.774	**0.250**
Cluster 1.1						193	13.000	11.670	48	2.240	0.534	0.759	**0.299**
Cluster 1.2						286	9.316	7.880	8	0.300	0.614	0.685	**0.105**
Cluster 2						421	11.158	11.160	21	0.002	0.452	0.737	**0.388**
Cluster 2.1						204	7.842	6.790	9	0.400	0.473	0.581	**0.188**
Cluster 2.2						76	7.842	8.470	3	0.250	0.518	0.726	**0.352**
Cluster 2.3						141	8.579	6.010	1	0.130	0.383	0.658	**0.276**

### Null alleles, Hardy-Weinberg equilibrium and linkage disequilibrium

The MICRO-CHECKER software [41] was used to check for potential scoring errors, large allele dropout and the presence of null alleles. The presence and frequency of null alleles were additionally examined using FreeNA [42] following the Expectation Maximization (EM) algorithm. The presence of null alleles may result in an overestimation of population differentiation. Thus, the same program was used to compute the *F*_ST_ statistic using and not using the *ENA* (Excluding Null Alleles) correction method. The bootstrap 95% confidence intervals (CI) for the global *F*_ST_ values were calculated using 50,000 replicates over loci. Pairwise linkage disequilibrium between loci and deviations from Hardy–Weinberg equilibrium (HWE) in each population and for each locus were checked using the software GENEPOP version 4.2.1 [43].

### Parameters of genetic diversity

Genetic diversity was measured for each sampling site as the mean number of alleles (Na), observed (H_O_) and expected heterozygosity (H_E_) and allelic richness (Ar) using FSTAT version 2.9.3.2 [44]. The observed number of private alleles were calculated with GDA [45], and a rarified measure of private allele richness (Par) was obtained with HP-RARE [46]. FSTAT was used to estimate the inbreeding coefficients of the populations (*F*_IS_) following Weir and Cockerham [47].

In order to visualize spatial patterns of genetic diversity, Ar, H_O_, H_E_ and Pa were spatially interpolated using ARCGIS 10.0 (ESRI, Redlands, CA) with the universal kriging function and a spherical semivariogram model [1]. Subsequently, in order to explore the correlation among the genetic diversity variables, a principal components analysis was conducted on the four genetic diversity variables with the XLSTAT software (Addinsoft, Paris). After checking for a positive correlation among these genetic variables, values for the first axis (PC1) were interpolated across the species’ range following the steps above.

### Genetic diversity across the geographic distribution range and distinct habitat types

Altitude and bioclimatic variables were downloaded from the WorldClim database version 1.4 (http://www.worldclim.org/) at a scale of 30 arc seconds (nearly 1 × 1 km, S1 Table). Slope and aspect were calculated from the altitude using ARCGIS. To avoid autocorrelation and over fitting of our data, collinearity among the initial 19 BioClim variables and the geographical variables (longitude, latitude, altitude, slope and aspect) was tested using the Pearson’s correlation coefficient in XLSTAT. A total of nine variables, all of which had a correlation degree lower than 0.75 (Pearson coefficient), were retained (S2 Table), i.e. four environmental variables and five geographical variables. A PCA was also conducted using the initial 19 BioClim variables and the geographical variables, which gave similar results (the same nine variables were choosen; data not shown). The final set of environmental predictor variables used consisted of: isothermality (BIO3), mean temperature of wettest quarter (BIO8), mean temperature of driest quarter (BIO9) and precipitation seasonality (BIO15); the final set of geographical variables were longitude, latitude, altitude, slope and aspect.

PCA was performed using XLSTAT to summarize and visualize the structure of data described by these nine (geographical and environmental) quantitative variables, using the genetic diversity variables of Ar, H_O_ and H_E_ as supplementary variables. This regression-based approach enables the quantitative estimation of genetic variation explained by environmental and geographical factors and their interaction effects. The relation between the different environmental variables and genetic variables and assignment was visualized by a biplot of the PCA on the environmental variables with plotted points colored according to their corresponding genetic cluster (see population structure section). Furthermore, to test the prediction that genetic diversity varied across the selected geographical and environmental gradients, univariate linear regressions were implemented between each of these genetic diversity measures (Ar, H_O_ and H_E_) and longitude, altitude and mean temperature of wettest quarter (BIO8). As most of the caves are located in the central-eastern part of the Pyrenees, and in order to exclude a possible interaction between longitude and habitat type, the correlation among longitude and altitude vs the genetic diversities was also measured by testing only streams. An ANOVA with Tukey HSD posthoc tests was used to compare the effect of the three distinct habitat types (streams, lakes and caves) to the main genetic diversity measures (Ar, H_O_ and H_E_). In the same way, as habitat types are unevenly distributed along the longitudinal axis, in order to avoid biased comparisons among habitat types due to their longitudinal localization, populations from the central Pyrenees (16 populations containing eight streams, five caves and three lakes) were considered and tested for effect of the three distinct habitat types as before.

### Population structure and differentiation

The pairwise population divergence between sampling localities was estimated with the *F*_ST_ as calculated in FSTAT and with Jost’s D [48] using the R package DEMEtics [49]. To assess the levels of population structure, several analyses were performed to avoid potential reliability issues of models and assumptions of certain software [50]. The level of genetic structure of *C*. *asper* populations was estimated using a Bayesian approach implemented in STRUCTURE version 2.3.4 [51]. Settings used included an admixture model with correlated allele frequencies, and the number of inferred clusters (*K*) ranged from one (complete panmixia) to 45 (i.e., the number of sample locations plus one). STRUCTURE was run for each value of *K* 10 times, with one million Markov Chain Monte Carlo (MCMC) iterations, discarding the first 10^5^ MCMC steps as burn-in phase. The optimal number of clusters was inferred using Δ*K* method by Evanno *et al*. [52], as implemented in STRUCTURE HARVESTER [53]. The average from all the outputs of each K was obtained with CLUMPP version 1.1.2 [54] and plotted with DISTRUCT version 1.1 [55]. In addition, we used POPTREEW [56] to construct a neighbor-joining tree using Nei’s genetic distance (D_A_, [57]) with 1000 bootstraps. An additional model independent clustering approach was performed using GENETIX, version 4.05.2 [58], by performing a factorial correspondence analysis on the allelic frequencies obtained for the 44 populations. This analysis was performed across the distribution range of *C*. *asper*, as well as for grouping the sampling localities as indicated by STRUCTURE.

Isolation by distance was evaluated by examining the relationship between geographic and genetic distances among populations with a Mantel test [59]. Geographic distances among populations were calculated and log-transformed to linearize the relationship between geographic distances and *F*_ST_ values (see [60]). Genetic distances were standardized as *F*_ST_/(1 − *F*_ST_), and the significance of matrix correlation coefficients was estimated in IBDWS (Web service; [61]) with 10,000 permutations. Analyses were performed between all sampled populations and by grouping the clusters.

In addition, the effective populations size (*N*_e_) for each population was calculated using two single-sample *N*_e_ estimators to check for convergent results: COLONY version 2.0.4.4 [62] and ONeSAMP [63]. COLONY takes into account possible genotyping errors and presence of null alleles and uses a maximum likelihood method to conduct sibship assignment analyses to estimate *N*_e_. COLONY was run using the maximum likelihood approach for a dioceous/diploid species, with medium length runs and both random and non-random mating, assuming polygamy for both males and females (as it is the case for most salamanders) with no sibship prior. We did not use the option ‘update allelic frequencies’ and other parameters used as default. ONeSAMP employs approximate Bayesian computation and calculates eight summary statistics to estimate *N*_e_ from a sample of microsatellite loci genotypes. The analyses were submitted online to the ONeSAMP 1.2 server (http://genomics.jun.alaska.edu/asp/Default.aspx). A variety of input priors were tested, with minimum *N*_e_ as low as 2 and maximum *N*_e_ as high as 1,000.

## Results

### Basic population genetic features and selection of microsatellite loci for further analysis

The highest percentage of null alleles was found for locus Ca16 in the population Barranco de Batisielles (BBA; 40%), while the average percentage was found to be 7.2% across the 44 populations analyzed. In total, null allele frequency estimates ranged from 0.3% to 7.2% with 2.5% on average across all loci. Global *F*_ST_ values calculated with and without correcting for null alleles had overlapping 95% confidence intervals (*F*_ST_ not using *ENA* = 0.3488 and *F*_ST_ using *ENA* = 0.3447 with the respective 95% CI [0.3023–0.4089] and [0.2985–0.4042]), which means that the impact of null alleles can be neglected. Classical measures of population differentiation are only slightly biased with a null allele frequency ranging between 5% and 8% on average across loci [42, 64]. Given that the average percentage of null allele frequency across loci (2.5%) is lower than 5%, and *F*_ST_ did not vary after excluding null alleles, all loci were kept for further statistical analyses.

Significant departures from HWE across loci were detected in 8 out of 44 (or 18%) sampling locations. Seven loci showed significant departures from HWE in one to three populations: Ca22 (two populations), Ca16 and Ca20 (three populations each), and Ca3, Us7, Ca5 and Ca25 (one population). These instances likely reflect occasional departures from random mating rather than the presence of null alleles. As none of the loci showed Hardy–Weinberg equilibrium deviations at more than three populations, we retained all loci in the analyses. Linkage disequilibrium was found only in Vidrà (VID) between five pairs of loci (Us7-Ca16, Us7-Ca30, Ca16-Ca25, Ca30-Ca25, Ca30-Ca29) after applying a Bonferroni correction (*p*<0.0003).

### Parameters of genetic diversity

Overall, values of observed and expected heterozygosity of *C*. *asper* (weighted average) were 0.521 and 0.523, respectively (Table 1). The mean number of alleles (Na) ranged from 1.58 to 8.21 (mean 4.28). The number of private alleles (PA) was generally low at a population level, most of the populations having none or one PA. Only Monrepòs (MON) and Sierra de Guara (SGU) had more than five private alleles. Allelic richness (Ar) varied across sites (1.44–4.50) with a mean of 2.89. Expected (H_E_) and observed heterozygosity (H_O_) ranged from 0.18 to 0.78 and from 0.18 to 0.72 respectively for all sites (mean H_E_ = 0.51, mean H_O_ = 0.52). In general, the most diverse populations in terms of Ar and H_E_ were MON and Sarvisé (SAR), both located at the south-eastern Pyrenees, while the least diverse populations were two cave populations, Betharram (BET) and Pas du Loup (PDL) (Table 1). Overall, *F*_IS_ was estimated to be 0.360 (*p*<0.005) but was not significantly different from zero for any population after applying Bonferroni’s correction (see Table 1). Significant *F*_IS_ values at the cluster level are probably caused by Wahlund effects and thus are probably not biologically relevant.

The first axis of the PCA (PC1) correlated positively with the genetic diversity variables (Ar, Par, H_O_ and H_E_) and accounted for 79.7% of their variance. The interpolated values of PC1 mapped into the species’ distribution range revealed a decrease of genetic diversity from the western Pyrenees to the eastern part (S1 Fig).

### Genetic diversity across the geographical distribution range

**Fig 2:**
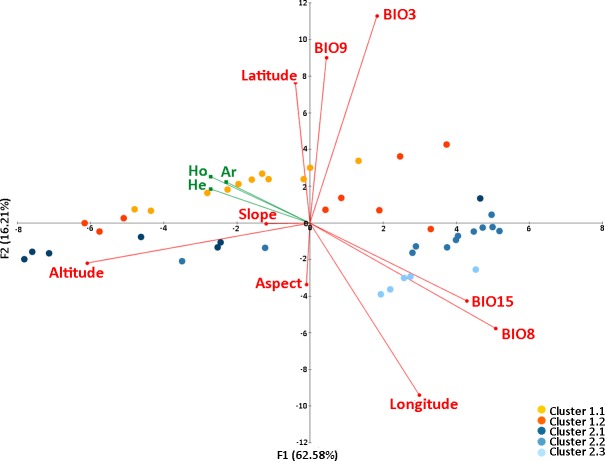
PCA plot based on nine environmental and geographical variables related to the genetic diversity. PCA plot based on four environmental variables and five geographical variables related to the genetic diversity measures (Ar, H_O_ and H_E_) describing the *Calotriton asper* populations.

The first two components of the PCA accounted for 78.79% of the total genetic variation among all the investigated populations (Fig 2). From the nine variables analyzed, only three (longitude, altitude and mean temperature of wettest quarter–BIO8) were significantly correlated with all genetic diversity parameters (S3 Table). From those, longitude and mean temperature of wettest quarter were negatively correlated with genetic diversity parameters while altitude was positively correlated (Fig 2).

**Fig 3:**
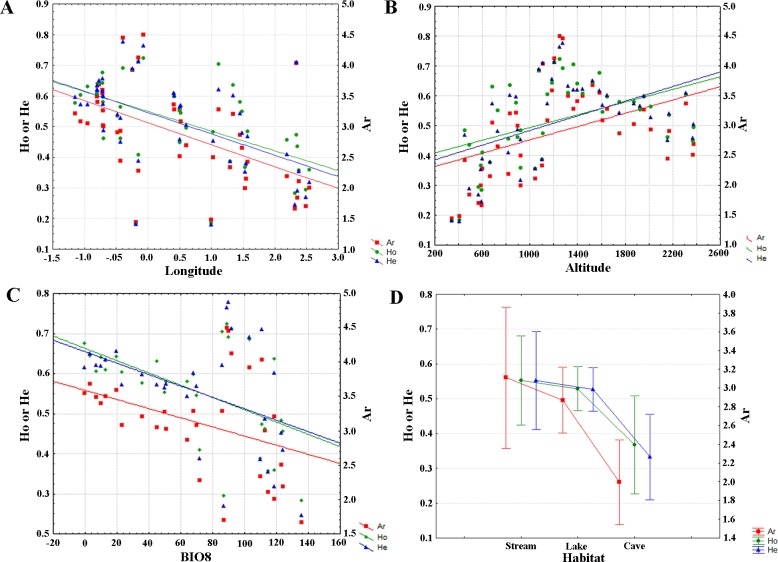
Linear regressions between the genetic diversity across the selected environmental and geographical variables. Linear regressions between the three genetic diversity indices (Ar, H_O_ and H_E_) across the longitude (A) and altitude (B) ranges, variable BIO8 (C) and the three distinct habitat types (D) of *Calotriton asper* populations.

The regression analysis shows a negative longitudinal and positive altitudinal gradient of genetic diversity in *C*. *asper* populations for the three genetic diversity measures analyzed (Fig 3, S3 Table). These patterns were also significant for stream populations alone (S2 Fig, S3 Table). Levels of genetic variation were consistently higher westwards and at high altitudes. This pattern has been also observed when only stream habitat localities were analyzed. When analyzed, the temperature-related variable (BIO8; mean temperature of wettest quarter) showed a similar negative correlation with genetic diversity as the variable longitude.

Genetic diversity was also significantly different in populations of different habitat type (S4 Table). However, Tukey HDS posthoc test showed that stream and lake populations had similar genetic diversity values while differences were due to caves. Cave habitats had significantly lower values of genetic diversity compared to streams or lakes. This pattern is also shown when only the central Pyrenees populations were analysed separatelly, i.e. even within the same longitudinal area caves were significantly poorer genetically than stream or lake populations.

### Population structure and differentiation

The mean *F*_ST_ value across all 44 sampling sites was rather high (0.304). Indeed, very high *F*_ST_ values were found in-between eastern and western populations of *C*. *asper*, reaching a maximum value of 0.771 for two separately located caves: Pas du Loup (PDL) and Betharram (BET; S5 Table). These two caves were the genetically most distinct populations found in our study. The former showed a *F*_ST_ of 0.267 with its closest locality Genie Longue (GLO), and a minimum of 0.401 with the remaining localities and a mean of 0.556. The minimum *F*_ST_ value between PDL and the other populations was 0.318 and the mean was 0.509. Most *F*_ST_ values (99% comparisons) were significant at *p* < 0.05 (S5 Table).

**Fig 4:**
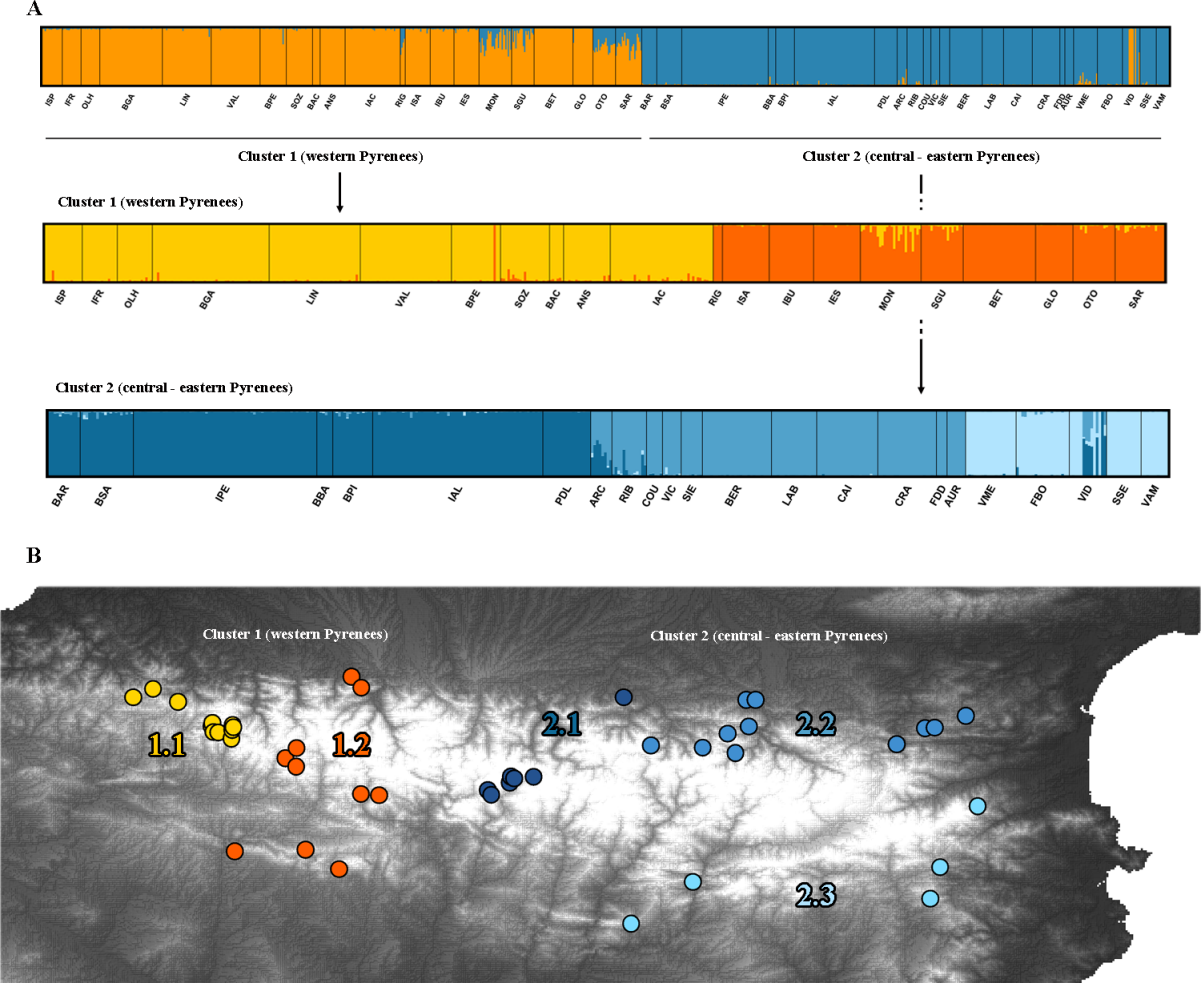
Genetic structure of the 44 *Calotriton asper* populations. Genetic structure of the 44 *Calotriton asper* populations. A) Results of Bayesian clustering and individual assignment analysis obtained with STRUCTURE at *K* = 2 including all *Calotriton asper* populations (above), the western Pyrenean populations with *K* = 2 and the central-eastern Pyrenean populations with *K* = 3. Each individual is represented by a thin bar corresponding to the sum of assignment probabilities to the *K* cluster. Black bars separate populations. B) Map of the five genetic clusters.

When analyzing the whole dataset, i.e. the 44 *C*. *asper* populations (*K*: 1 to 45), a clear peak of delta*K* was detected at *K* = 2 (S3 Fig). Populations from the western Pyrenees were assigned to the same cluster while populations from the central-eastern Pyrenees were clearly grouped apart (Fig 4). When we analyzed both clusters separately, the western populations (cluster 1) remained grouped into two clusters (*K* = 2; S3 Fig): one contained the westernmost populations, while the other grouped together with the western populations at both sides of the Pyrenees. When analyzing cluster 2 (the central-eastern Pyrenees) a clear peak of *K* = 3 (S3 Fig) was obtained. The most central-eastern populations were assigned to the same cluster (cluster 2.1) while populations from north-eastern and south-eastern Pyrenees were clearly grouped apart into two groups (clusters 2.2. and 2.3).

Although the number of private alleles was low at a population level, clusters showed a high number of private alleles, being much higher in cluster 1 than in cluster 2 (PA = 72 and 21, respectively; Table 1). Subcluster 1.1. was the most differentiated one accounting for 48 private alleles, while other subclusters presented less than 10 private alleles. Allelic richness (Ar) was higher in the western populations (cluster 1) with subcluster 1.1 being the richest, while the central-eastern populations (cluster 2) were poorer with subcluster 2.3 being the poorest. Expected (H_E_) and observed (H_O_) heterozygosity were also higher in western populations than central-eastern ones (Table 1). The *F*_ST_ values were all significantly different between the Pyrenean clusters and ranged from 0.146 to 0.277 (Table 2). The lowest *F*_ST_ values were found between clusters 1.1 and 1.2 (western Pyrenees). Instead, within eastern Pyrenees (clusters 2.1, 2.2 and 2.3) higher values of *F*_ST_ were found (0.262–0.277). The neighbor-joining tree recovered the five groups identified by STRUCTURE (S4 Fig).

**Table 2:** Pairwise *F*_ST_ values among *Calotriton asper* genetic clusters. Pairwise *F*_ST_ values among *Calotriton asper* genetic clusters by STRUCTURE analysis. All *p*<0.0001.

	Cluster 1		Cluster 2		
	1.1	1.2	2.1	2.2	2.3
**1.1**	-				
**1.2**	0.14623	-			
**2.1**	0.23406	0.24094	-		
**2.2**	0.21594	0.27496	0.27678	-	
**2.3**	0.23311	0.23467	0.26227	0.26828	-

**Fig 5:**
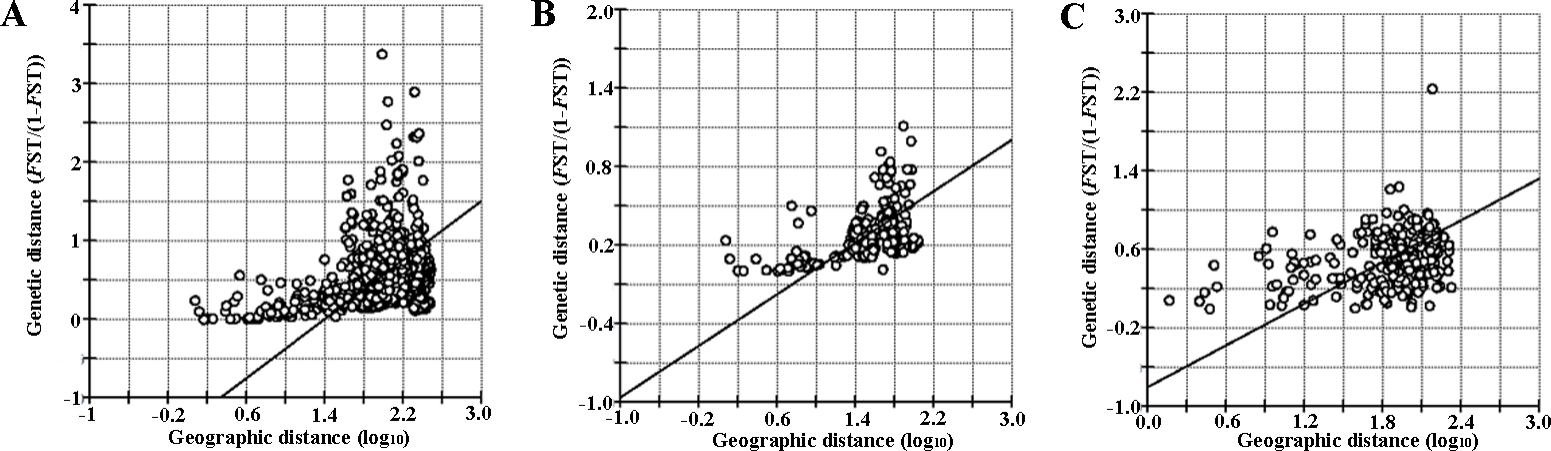
IBD for the total dataset and within clusters. Isolation-by-distance for the total dataset (A), within cluster 1 (B) and within cluster 2 (C). Geographic distance is log-transformed and genetic diversity is standardized as *F*_ST_/(1 − *F*_ST_).

Mantel tests of isolation by distance (IBD) over all populations revealed a moderated but significant (*p* < 0.001), positive relationship between geographical and genetic distances (*R*^2^ = 0.112; S6 Table, Fig 5). The increase of isolation with geographical distance is consistent with the population structure detected from the Bayesian clustering analysis (see Structure analyses). A similar result was found when analyzing the cluster 1 and 2 separately (*R*^2^ = 0.197 and *R*^2^ = 0.102, respectively, *p* < 0.001).

The two methods used to estimate the effective population sizes (*N*_e_) resulted, in general, in low values (see S7 Table). Considering both methods, effective population sizes ranged between four and 306. Barbarisa (BAR) and Oto (OTO) showed values over 200 using COLONY, and Ibón de Perramó (IPE) showed values over 100 with ONeSAMP. The remaining populations showed estimates below 100, with a mean *N*_e_ of 21–56 (S7 Table).

## Discussion

Here, we have analyzed a large genetic data set of the endemic Pyrenean brook newt *C*. *asper* to investigate impacts of glacial isolation on the population structure in a mountain context. We found a positive correlation between genetic diversity and altitude, which appears to be generally rare, as most commonly negative correlations are observed. We also found an unusually high level of genetic differentiation as expressed by *F*_ST_ values among the Pyrenean brook newt populations. These values are the highest ever found for intraspecific comparisons of an amphibian species so far (e.g. [19] and references herein). Interestingly, these high levels of nuclear genetic divergence contrast with a relatively low mitochondrial diversity of *C*. *asper* populations (see [32]), which suggests a relatively recent shared population history of *C*. *asper*. However, clear genetic differences between hypogean and epigean populations suggest a long isolation of these populations.

### Genetic variability, longitudinal and altitudinal patterns across the Pyrenees

Microsatellite loci analysis revealed similar levels of genetic variability in *C*. *asper* populations across the Pyrenees (weighted average Ar = 2.930 and H_E_ = 0.523) compared to its sister species, the Montseny brook newt, *C*. *arnoldi* (weighted average Ar = 3.398 and H_E_ = 0.441)[26]. These values are also consistent compared to other mountain brook newts, such as *Euproctus platycephalus* on Sardinia (weighted average Ar = 2.376 and H_E_ = 0.6 [65]) and are within the typical range of other urodeles and temperate amphibians (0.4–0.6; [66] and references therein). In accordance with other Pyrenean species, such as the Ericaceae *Rhododendron ferrugineum* [8], we found that genetic diversity of *C*. *asper* populations is negatively correlated with longitude. The western populations were in general more genetically diverse than those in the central or eastern areas of the Pyrenees. In fact, climate across the Pyrenees varies greatly due to oceanic influences in the west and Mediterranean influences in the east of the chain [8]. Parts of the Pyrenees that are influenced by the Mediterranean climate provide higher temperatures and drier conditions than oceanic influenced sectors, which provide more suitable habitats for *C*. *asper* [28]. Moreover, a western evolutionary origin remains possible as a Pleistocene fossil assigned to *Calotriton* has been found in the Cave of the Hyenas (Asturias) [67, 68].

The most striking result concerning genetic variability in our study was the positive correlation with altitude, i.e. genetic diversity (in terms of allelic richness and expected heterozygosity) tended to be higher at high-altitude populations. In fact, maximum values can be observed at an altitudinal range around 1,200 m, but levels still remained high at high-altitude populations (e.g. in Barbarisa at 2,333 m: Ar = 0.360 and H_E_ = 0.609; Table 1). The impact of altitudinal gradient on the genetic patterns differs between amphibian species. In some species there is no correlation found between the altitudinal gradient and the population differentiation or genetic diversity (e.g. *Rana chensinensis* [69]; *Euproctus platycephalus* [65]). In other species, the genetic diversity is negatively correlated with altitude (e.g. *Rana luteiventris* [70]; *Ambystoma macrodactulym* [71]). However, to the best of our knowledge, our results represent the first case of a positive correlation between genetic diversity and an altitudinal gradient (Fig 3B). Again, *C*. *asper* seems to prefer cooler and wetter environments at high altitudes than warmer and drier valleys.

Mountains have repeatedly been shown to have strong effects on gene flow in amphibians [72]. Considering this fact, we wonder if the top hill Pyrenees imposes a genetic barrier to the *C*. *asper* populations. Populations at the periphery of a species' distribution tend to have lower genetic diversity than the central populations probably attributable to suboptimal habitat, greater isolation, founder effects and/or genetic bottleneck [69, 73]. By occupying different habitats, such as streams, lakes and caves [28], environmental plasticity or the ability to locally adapt to different habitat conditions–or both mechanisms–appear to be high in *C*. *asper*. Given that *C*. *asper* can be generally seen as a typical cold-adapted mountainous freshwater amphibian species with an optimal elevation range between 1,000–2,000 m asl [28], habitats in the lowlands with high water temperatures should be less suitable for this species. We therefore assume that gene flow will most likely occur through the most suitable habitats at higher elevations. This is in accordance to the predicted distribution models inferred for the interglacial periods (warm periods), when this species would have been expelled from the lower areas (i.e. < 500 m; [11]). Along this line, the high but not maximum genetic variability at high-altitude sites may also be interpreted as a consequence of a more recent colonization of high altitude habitats located around 1,500 m asl of the Pyrenees after the last glaciation events (LGM, 20000 ya). Within our dataset, five out of six localities below 500 m asl are caves, while lakes represent the most elevated sites above 1,800 m asl. Although habitat type might be affecting the genetic variability (see discussion below), altitude showed a significant positive correlation when only stream populations were analyzed (see S2 Fig and S3 Table).

We therefore conclude that the highest levels of genetic diversity of stream populations of *C*. *asper* are found around 1,200 m asl, and the lowest below 700–1,000 m asl. However, regarding genetic diversity levels at high elevations and the cluster structure assembling French and Iberian populations (clusters 1.2 and 2.1), we can assert that high altitude mountains may not be acting as a dispersal barrier in *C*. *asper*. There might be enough passages at high altitudes that are suitable for migration during the summer period where temperatures are not too low. Some of the migration, however, will likely occur horizontally in a stretch of a suitable mountain region. Thus, French and Iberian high altitude populations may be genetically connected, but lowlands at both sides of the Pyrenees may constitute a harsh habitat with peripheral and isolated populations.

### Patterns of genetic diversity between distinct habitat types

The environmental plasticity previously mentioned for this species matches with similar levels of genetic diversity found between lakes and streams. Streams are described as the ‘optimal’ habitat for this species, and high mountain lakes and caves have been seen as secondary habitats [28]. Our results in terms of genetic diversity and the number of private alleles suggest that newts that colonized high mountain lakes had been quite successful. Despite being posteriorly colonized areas (lakes had lower number of private alleles than streams), lakes maintain similar levels of genetic variation as streams. Caves are generally characterized by food restriction [38]. However, they represent a valuable habitat for amphibians as they can hold water all year round and therefore can also favor the survival of larvae and metamorphosed individuals [74, 75]. Moreover, in our case, caves were located in lowlands where epigean water and air temperature might be too high. We assume that *C*. *asper* uses caves in the lowlands as a substitute habitat for cold-water streams and individuals became enclosed and isolated from nearby populations. It seems that newts from streams might be dispersing when they ‘fall into the caves’ or enter voluntarily when conditions outside are harsher. The low levels of genetic variability together with the almost absence of private alleles (only in one of the caves) suggested a secondary colonization of lowland caves from neighboring populations rather than being rear-edge remnant populations left from the lowland refugia [8, 76]. In fact, some authors suggest that this colonization occurred rather recently only 10,000 ya [38, 74].

### Genetic structure of *C*. *asper* populations

Overall, microsatellite loci data support reduced gene flow even among some neighboring populations together with a strong genetic structure and a moderate signal of isolation-by-distance. The extremely high values of *F*_ST_ found within *C*. *asper* populations are the highest ever found in similar species, considering the revision of Chan and Zamudio [66] who compared 16 temperate amphibians. Another ecologically similar mountain newt, *Euproctus platycephalus* endemic to the island Sardinia (Italy) presents a maximum level of pairwise population differentiation considerably lower than *C*. *asper* (0.297 among two localities separated by 168 km; [65]). Lower *F*_ST_ values were also found for the long-toed salamander *Ambystoma macrodactylum* (*F*_ST_ of 0.27 [19]).

Philopatry and dispersal capability, two linked biological attributes of a species, have a great influence on the population structure and isolation. For *C*. *asper*, a 2-year-long terrestrial juvenile dispersal phase was described [30], providing the opportunity to explore and colonize new habitat niches. The fact that *C*. *asper* inhabits high-altitude lakes and streams in areas that were recently glaciated, has been also seen as evidence for a high dispersal capacity [32, 77]. Other stream salamanders were also shown to be able to use both aquatic and overland dispersal [78]. However, results from capture-recapture studies [31] advocated that the dispersal capability during the terrestrial phase may actually be limited in *C*. *asper*. Accounting for the extremely high values of genetic differentiation found among their populations–similar to what was found with AFLPs [32]–the low dispersal capability for this species is reinforced. In fact, the dispersal capability of species is not exclusively determined by its intrinsic biology but also by extrinsic factors such as landscape characteristics, climatic suitability, and water quality [18, 19]. The external factors may be more remarkable in mainly aquatic species, as is the case of *C*. *asper*, as dispersal may be strongly influenced by the presence of surrounding suitable corridors (a stretch of optimal habitat that facilitates the migration of individuals, see introduction section). In an optimal environment, these species may easily migrate from site to site through present corridors. Instead, in populations surrounded by less suitable habitats, migration might be hampered [79]. The most diverse populations in our study (mainly cluster 1.1. but also 1.2; Table 1) but less differentiated are the ones located westwards (*F*_ST_ within cluster 1 of 0.146; Table 2). These populations may be more connected by suitable habitat corridors, enabling an easier dispersal of individuals between sites. Occasional gene flow among localities may, in turn, support the maintainance of high levels of genetic diversity [18, 19, 35, 80]. Instead, central and south-eastern populations (clusters 2.1 and 2.3) present higher values of population differentiation (*F*_ST_ around 0.270; Table 2) and are the poorest in terms of genetic diversity (Table 1).

### Inferring a biogeographic scenario for *C*. *asper*

A biogeographic scenario inferred for *C*. *asper* also matches with that of other mountain organisms [35, 81] as well as other Pyrenean distributed species, such as spiders or plants [8, 9]. Regarding mitochondrial markers, *C*. *asper* is not clearly structured across its distribution range and the low divergence suggests a recent ancestry of *C*. *asper* populations. In contrast, concerning microsatellite loci differentiation, this species is clearly structured into two main and several subclusters across the Pyrenees (see Fig 4). The structure into two genetically distinct groups across the Pyrenees is not exclusive for this species; in fact, the different lineages in animals and plants found in this mountain range most often observed is two [9, 81, 82], separated across the longitudinal axis (eastern and western lineages). Studies that found the two main lineages argue that species may have survived glaciations in two main refugia–one in south central and another in the eastern Pyrenees. In our case, the unclear structure and the low genetic diversity at the mitochondrial level together with the predicted distribution models inferred for the LGM [11, 32, 33] may be interpreted as an intense past gene flow between populations at lowland and midland refugia, i.e. populations would have come into contact, exchanging genes and homogenizing [77, 83, 84]. However, the genetic signal (genetic diversity patterns and population structure) found with microsatellite loci would fit with the recent recolonization of the high mountains during the interglacial periods. Genetically connected populations would expand from large lowland refugia to high altitude plateaus [8]. Subsequently, these large high altitude populations could have been fragmented when the climate became warmer and drier–especially intense in some areas (e.g. deep valleys). In fact, central-eastern populations may have been isolated after the recolonization due to the absence of corridors (stronger differentiation between their populations). Instead, current gene flow within western populations seems more likely to occur (lower differentiation between clusters) due to the presence of suitable corridors. High numbers of lineages in the Pyrenees are also shown, e.g. for *Rhododendron ferrugineum* with five clusters [8] fitting to the clustering of *C*. *asper* into five groups.

### Implications for conservation

Regarding the genetic diversity and population structure of *C*. *asper* shown in our study, the most vulnerable areas are those at the limit of its distribution, such as the eastern Pyrenees and/or the lowlands, basically due to the less suitable environmental conditions. Taking into account this scenario, future predictions of climate change may drastically reduce the potential distribution range of this species [85]. The mountain critical zone at mid-altitudes may see important climatic changes over time and are already under high anthropogenic pressure due to pastorialism and tourism. Hence, *C*. *asper* might get trapped in high mountain habitats due to global change. However, as it is the case for many mountain lakes across the world [86, 87], Pyrenean lakes and their biota suffer from fish introductions and natural colonisations may occur only when concrete management actions are put in place [88, 89].

Caves as habitats for *C*. *asper* are needed to be treated with caution due to their isolation and the low levels of genetic diversity. Also, some highly diverse stream populations, such as those of Monrepós (MON) and Sierra de Guara (SGU), presenting high allelic richness and a high number of private alleles, should be considered as populations of interest for conservation, as they harbor a large proportion of the species’ genetic diversity.

Except from Barbarisa (BAR), Oto (OTO) and Ibón de Perramó (IPE), the remaining populations showed low effective population sizes (*N*_e_ < 100). An effective population size of 500 has been suggested as a minimum value for the long-term survival of a species, and values around 50 in isolated populations are considered of major concern [90]. However, values of *N*_e_ around 50 are not unusual for amphibians [91]. Effective population sizes for the *C*. *asper* populations were similar or higher than those of the *C*. *arnoldi* populations or other urodele amphibians [26]. Here, nine of the 44 *C*. *asper* populations analyzed had an *N*_e_ lower than 10. Populations with a very low *N*_e_ are needed to be considered of particular concern, as these populations have an increased probability of extinction resulting from genetic effects, such as inbreeding [92] and stochastic environmental processes.

Because of the differentiation between populations highlighted in this study, it is essential for management or possible re-introduction programs to take into account the results of molecular studies [93]. The influence of a high pressure of interspecific competition and predation (e.g. by invasive fish) imposes a recent serious threat to the conservation of this species. In fact, *C*. *asper* has been experiencing a substantial reduction of its habitat [88, 89] and accordingly, management measures are needed to reduce the pressure of interspecific competition and predation (e.g. by invasive fish). Particularly, isolated and fragmented populations may need to be managed independently [35].

## Supporting information

S1 Table List and codification of environmental variables from the BIOCLIM dataset. List and codification of environmental variables from the BIOCLIM dataset. Units expressed and mean, median, minimum and maximum values for the 44 *Calotriton asper* populations. Temperature data is in units °C *10 with a 0.1°C precision.
(XLSX)

S2 Table Pearson correlation matrix of environmental and geographical variables. Pearson correlation matrix of environmental and geographical variables. In bold, significant levels of *p*<0.05. See text, for the codes of the variables.
(XLSX)

S3 Table Univariate linear regression between genetic diversity and geographical and environmental selected variables. Univariate linear regression between genetic diversity and geographical and environmental selected variables of *Calotriton asper* populations. In bold significant levels of *p*<0.05.
(XLSX)

S4 Table ANOVA among the three distinct habitat types to genetic diversity. ANOVA with Tukey HSD posthoc test among the three distinct habitat types to allelic richness (Ar) observed (H_O_) and expected heterozygosity (H_E_).
(XLSX)

S5 Table Pairwise *F*_ST_ values among *Calotriton asper* populations. Pairwise *F*_ST_ values among *Calotriton asper* populations. See Table 1 for the population codification; *p* values were <0.05 for all pairs except for those in bold.
(XLSX)

S6 Table IBD of the five genetic clusters. Results of the isolation by distance analyses (IBD; Mantel test) of the five genetic *Calotriton asper* clusters.
(XLSX)

S7 Table Estimates of effective population size (*N*_e_) for each *Calotriton asper* population. Estimates of effective population size (*N*_e_) for each *Calotriton asper* population, calculated with two analytical approaches (ONeSAMP, and COLONY) and using different priors; estimates of the upper and lower 95% CI estimates for each method are indicated.
(XLSX)

S8 Table Microsatellite data of the *Calotriton asper* dataset used. (XLSX)

S1 Fig Spatial interpolation of the genetic diversity measures acroos the *Calotriton asper* distribution range. Spatial interpolation of the genetic diversity of *Calotriton asper*. A) First axis of a principal components analysis (PC1) summarizing the allelic richness, the rarified private allele richness and the observed and expected heterozygosities (positive PC1 scores indicate higher diversity). B) Expected heterozygosity, H_E_. C) Observed heterozygosity, H_O_. D) Allelic richness, Ar. E) Rarified private allele richness, Par.
(TIF)

S2 Fig Linear regressions between the genetic diversity across the longitude and altitude using only stream populations. Linear regressions between the three genetic diversity indices (Ar, H_O_ and H_E_) across the longitude (A) and altitude (B) ranges when only stream *Calotriton asper* populations were analyzed.
(TIF)

S3 Fig Plot of delta*K* values calculated by Evanno’s method from the Structure analyses. Plot of delta*K* values calculated by Evanno’s method from the Structure analyses. A) Global dataset of the 44 *Calotriton asper* populations. B) Western Pyrenean populations, i.e. Cluster 1. C) Central-eastern Pyrenean populations, i.e. Cluster 2.
(TIF)

S4 Fig Neighbor-joining tree among the *Calotriton asper* populations. Neighbor-joining tree using D_A_ distances among the *Calotriton asper* populations showing the relationships between the five genetic clusters defined by STRUCTURE analysis. As for the population codifications see Table 1.
(TIF)
