# Supplementary figures and images for: Jailed in the mountains: Genetic diversity and structure of an endemic newt species across the Pyrenees
Source: PLoS One. 2018 Aug 2;13(8):e0200214. doi: 10.1371/journal.pone.0200214 (PMC6071966; doi:10.1371/journal.pone.0200214)

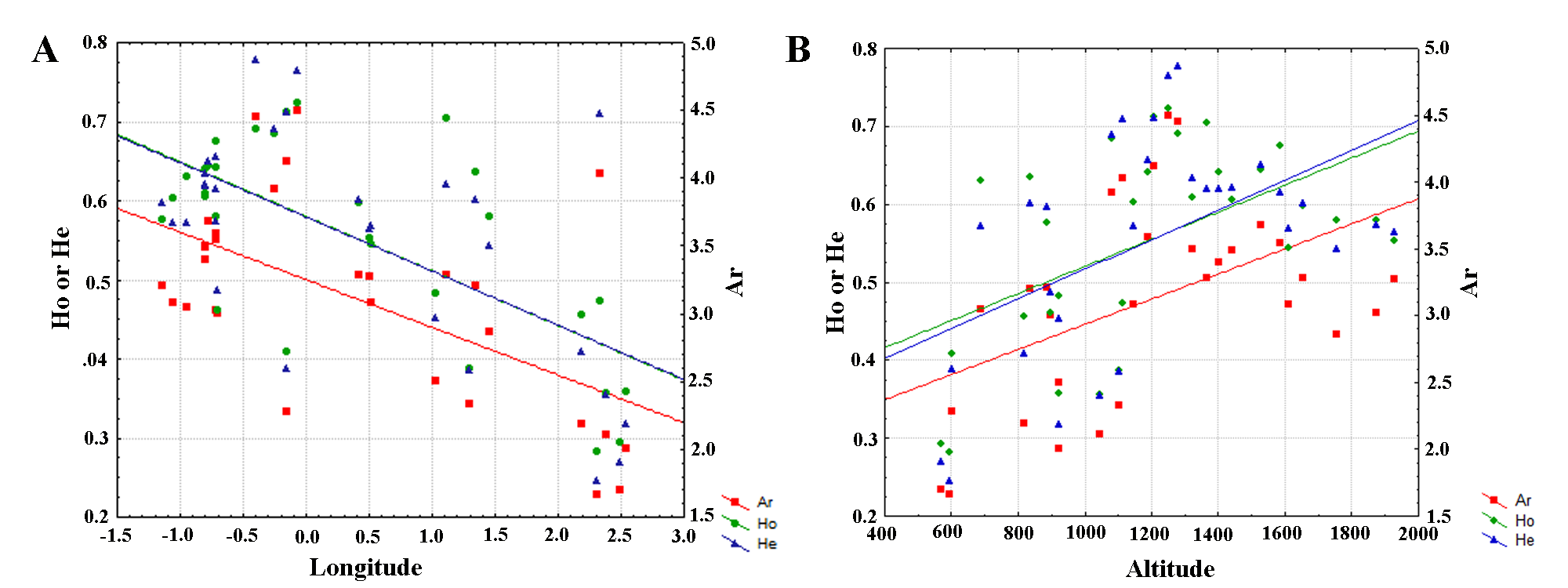

Supplement: S2 Fig — Linear regressions between the three genetic diversity indices (Ar, HO and HE) across the longitude (A) and altitude (B) ranges when only stream Calotriton asper populations were analyzed. (TIF) [file pone.0200214.s010.tif]

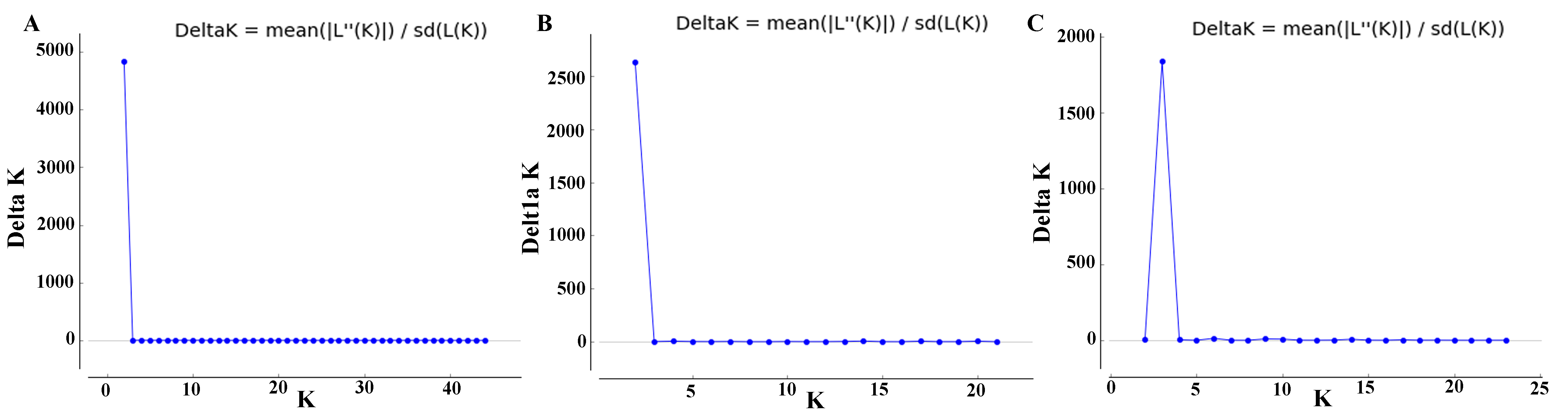

Supplement: S3 Fig — Plot of deltaK values calculated by Evanno’s method from the Structure analyses. A) Global dataset of the 44 Calotriton asper populations. B) Western Pyrenean populations, i.e. Cluster 1. C) Central-eastern Pyrenean populations, i.e. Cluster 2. (TIF) [file pone.0200214.s011.tif]

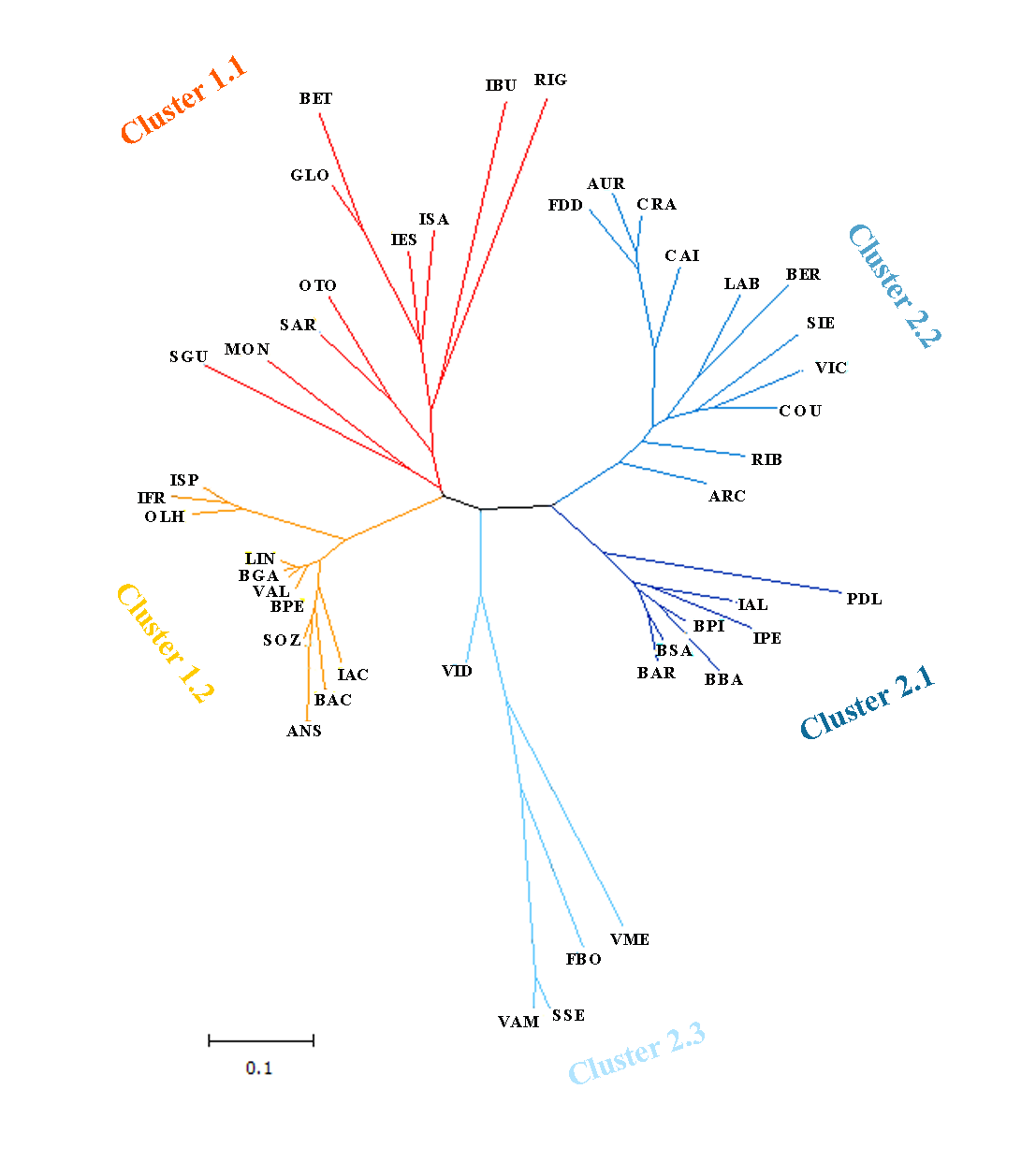

Supplement: S4 Fig — Neighbor-joining tree using DA distances among the Calotriton asper populations showing the relationships between the five genetic clusters defined by STRUCTURE analysis. As for the population codifications see Table 1. (TIF) [file pone.0200214.s012.tif]
